# Pharmacological Inhibition of HDAC6 Attenuates NLRP3 Inflammatory Response and Protects Dopaminergic Neurons in Experimental Models of Parkinson’s Disease

**DOI:** 10.3389/fnagi.2020.00078

**Published:** 2020-03-31

**Authors:** Shaoqi Yan, Xinbing Wei, Wencheng Jian, Yue Qin, Jia Liu, Shaowei Zhu, Fan Jiang, Haiyan Lou, Bin Zhang

**Affiliations:** ^1^Department of Pharmacology, School of Basic Medical Sciences, Shandong University, Jinan, China; ^2^Department of Radiology, Qilu Hospital, Shandong University, Jinan, China; ^3^Department of Neurology, Qilu Hospital, Shandong University, Jinan, China; ^4^Department of Physiology and Pathophysiology, School of Basic Medical Sciences, Shandong University, Jinan, China; ^5^Key Laboratory of Cardiovascular Proteomics of Shandong Province, Qilu Hospital, Shandong University, Jinan, China

**Keywords:** HDAC6, NLRP3, Parkinson’s disease, peroxiredoxin 2, tubastatin A

## Abstract

**Aim:**

To investigate the role of histone deacetylase 6 (HDAC6) deacetylation activity in nucleotide-binding oligomerization domain and leucine-rich repeat pyrin 3 domain (NLRP3) inflammatory response and explore the effects of pharmacological inhibition of HDAC6 with tubastatin A (TBA) on dopaminergic injury.

**Methods:**

Using 6-OHDA-induced Parkinson’s disease (PD) models, we examined the effects of TBA on NLRP3 activation and cell injury in SH-SY5Y cells. We also investigated the effects of TBA on NLRP3 inflammatory responses and dopaminergic injury in the nigrostriatal system in mice and analyzed the acetylation levels of peroxiredoxin2 (Prx2) and oxidative stress.

**Results:**

TBA inhibited 6-OHDA-induced NLRP3 activation, as demonstrated by decreased expressions of NLRP3 and matured caspase-1 and IL-1β, and also alleviated glial proliferation and dopaminergic neuronal degeneration. Notably, TBA recovered acetylation levels of Prx2 and reduced oxidative stress.

**Conclusion:**

Our findings indicate that pharmacological inhibition of HDAC6 with TBA attenuates NLRP3 inflammation and protects dopaminergic neurons, probably through Prx2 acetylation. This study suggests that the deacetylase catalytic domain of HDAC6 is a potential target for PD treatment.

## Introduction

Parkinson’s disease (PD), a chronic and degenerative neurological disorder caused by dysfunction of the extrapyramidal system, is characterized by loss of dopaminergic neurons in the substantia nigra pars compacta (SNpc) and striatum (STR) (Hauser and Hastings, 2013; Pan et al., 2018). Multiple pathogenic mechanisms have been demonstrated to be involved in PD, and there is currently no cure. Inflammatory reactions have been shown to be critical for the development of PD (Calabrese et al., 2018; Stephenson et al., 2018), and increased production of inflammatory cytokines such as interleukin (IL)-1β and IL-18 can be found in the postmortem brains in PD patients (Joshi and Singh, 2018). Notably, anti-inflammatory drugs have shown to be effective in the treatment of PD (Kurkowska-Jastrzebska et al., 2004; Gagne and Power, 2010). Hence, neurogenic inflammation may be a potential therapeutic target for preventing PD.

Histone deacetylase 6 (HDAC6), one of the class II HDAC family, is regarded as an unusual HDAC due to its unique properties: it is mainly located in the cytoplasm and contains two independent deacetylase catalytic domains (DD1 and DD2) and a zinc finger ubiquitin-binding domain (ZnF-UBP) (Simoes-Pires et al., 2013; Liang and Fang, 2018). DD1 and DD2 contribute to the enzymatic activity of HDAC6 (i.e., deacetylation activity), and ZnF-UBP exerts non-enzymatic effects (i.e., ubiquitin-binding activity) (Du et al., 2010). Accumulating studies have demonstrated that pharmacological inhibition of HDAC6 promotes Treg suppressive activity in models of autoimmunity and inflammation (de Zoeten et al., 2011), and the HDAC6 selective inhibitor MC2780 inhibits host inflammation in silicone implants by reducing thickness of peri-implant fibrous capsule and production of IL-1β (Di Liddo et al., 2016); in addition, inhibition of HDAC6 with ACY1083 protects intestine by attenuating inflammation during hemorrhagic shock (Chang et al., 2018), and pharmacological inhibitors of HDAC6-treated mice are protected from inflammatory responses caused by methicillin-resistant *Staphylococcus aureus* (Karki et al., 2019). Therefore, these studies indicate that HDAC6 inhibitors, which target deacetylase catalytic domains only (Miyake et al., 2016), exhibit anti-inflammatory properties in various pathologic conditions.

However, the effects of selective HDAC6 inhibitors on PD development have not yet been characterized. Compelling evidence indicates that HDAC6 may be relevant to inflammatory immune response, suggesting the potential influence of HDAC6 on PD-associated inflammation and the potential application of HDAC6 selective inhibitors in PD. Among multiple inflammatory mediators, nucleotide-binding oligomerization domain and leucine-rich repeat pyrin 3 domain (NLRP3) may have an important role in the pathogenesis of PD. NLRP3 is an intracellular mediator that can trigger an inflammatory cascade in response to cellular stress (Martinon et al., 2002). Once activated, NLRP3 would oligomerize to form inflammasome and promote maturation of caspase-1 and several proinflammatory cytokines including IL-1β and IL-18 (Shen et al., 2018). Although NLRP3 inflammasome signaling has generally been considered an immune cell-related inflammatory process (Zhou et al., 2016; Lee et al., 2018; Voet et al., 2018; Houtman et al., 2019), expression and activation of inflammasome components have also been reported in neurons (Ye et al., 2017; Teng et al., 2018; Cowie et al., 2019; Pronin et al., 2019); notably, Zhang et al. (2016) demonstrated that neuronal activation of NLRP3 is involved in the pathogenesis of PD. However, the potential mechanisms regulating NLRP3 activity in PD are poorly understood. Considering the anti-inflammatory properties of HDAC6-specific inhibitors, this study investigated the effects of pharmacological inhibition of HDAC6 by tubastatin A (TBA), an HDAC6 selective inhibitor, on activation of NLRP3 inflammatory response and characterized the effects of TBA on PD pathogenesis in *in vitro* and *in vivo* PD models.

## Materials and Methods

### Cell Culture

**FIGURE 1:**
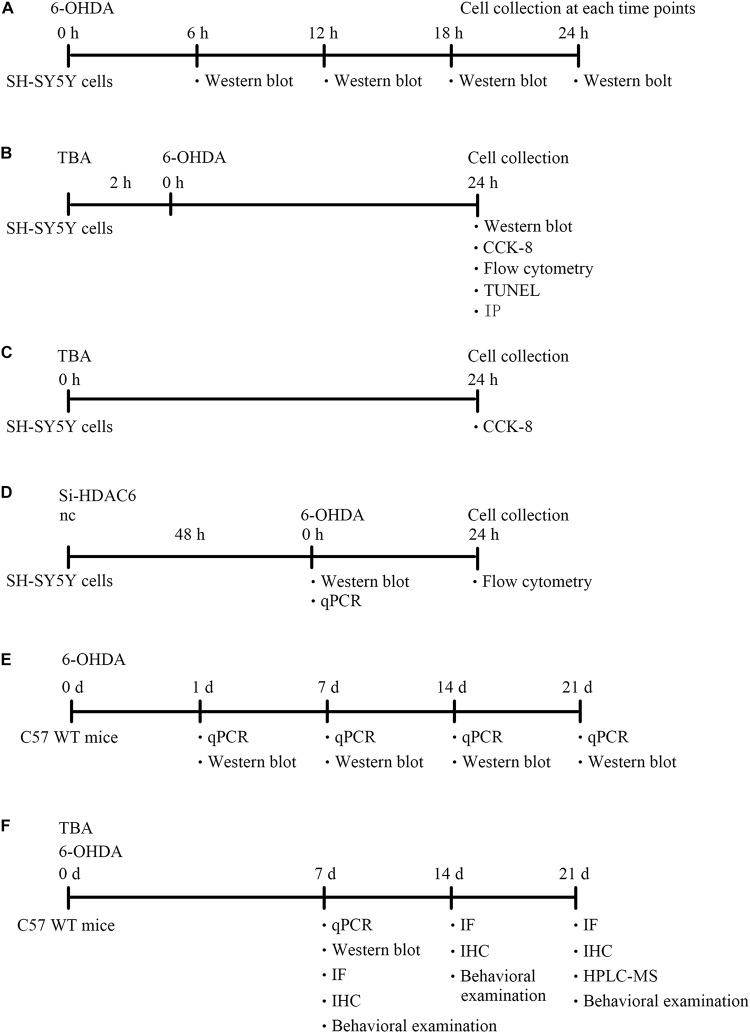
A diagram of the experimental design (timeline). **(A)** Experimental design for evaluation of NLRP3 expression in SH-SY5Y cells after 6-OHDA injury. **(B)** Experimental design to explore the role and mechanism of TBA in SH-SY5Y cells after 6-OHDA injury. **(C)** Experimental design to explore the effects of TBA on cell viability in SH-SY5Y cells. **(D)** Experimental design for evaluation of HDAC6 expression in siRNA-HDAC6-transfected SH-SY5Y cells and the effects of siRNA-HDAC6 on cell apoptosis after 6-OHDA injury. **(E)** Experimental design for evaluation of NLRP3 expression in 6-OHDA-induced PD mouse model. **(F)** Experimental design to explore the role and mechanism of TBA in 6-OHDA-induced PD mouse model.

The human neuroblastoma cell line SH-SY5Y was cultured at 37°C in DMEM-F12 supplemented with 10% fetal bovine serum in a humidified atmosphere of 5% CO_2_. Cells were pretreated with TBA (S8049, Selleck) for 2 h and were then subjected to 6-OHDA stimulation for different hours (6, 12, 18, and 24 h). siRNA-HDAC6 (NM_006044, Sigma-Aldrich) was also used in this study. The diagram of the design for cell experiments were shown in Figures 1A–D.

### Animal Surgery

All animal experiments were pre-approved by the Institutional Animal Care and Use Committee of Shandong University. Male C57BL/6 mice (8 weeks old) were anesthetized with 30 mg/kg sodium pentobarbital and placed in a stereotaxic device, and 9 μg of 6-OHDA (162957, Sigma-Aldrich) was then injected into two different sites of the right STR of the brain using a Hamilton syringe. The stereotaxic coordinates of the two different sites of the right STR, measured in millimeters, were as follows: bregma + 1.0, lateral 2.1, and ventral −2.9 and bregma −0.3, lateral 2.3, and ventral −2.9. Mice were decapitated for biochemical or histological assessments at different time points (1, 7, 14, and 21 days) following 6-OHDA injection. For TBA treatment, mice were administered 25 mg/kg TBA (intraperitoneal injection) for 7 days after 6-OHDA exposure. The diagram of the design for animal experiments were shown in Figures 1E,F.

### Western Blotting

Protein extracts were prepared from SH-SY5Y cells or brain tissues by homogenization in RIPA buffer with protease inhibitors and then were centrifugated to eliminate particulates at 14,000 *g* for 10 min at 4°C. Protein concentration was measured by the BCA method. Samples were separated on SDS-PAGE and transferred to nitrocellulose membranes. Immunoblotting was carried out with the primary antibodies specific for NLRP3 (ab4207, Abcam), caspase-1 (ab1872, Abcam), IL-1β (16806-1-AP, Proteintech), HDAC6 (07-732, Millipore), tyrosine hydroxylase (TH; MAB318, Millipore), acetylated lysine (9441, Cell signaling), peroxiredoxin 2 (Prx2; ab109367, Abcam), and 4-Hydroxynonenal (4-HNE; ab46545, Abcam). The primary antibody against β-actin (CW0096, CWBIO) was used to correct the sample loading. Densitometry analysis was performed using AlphaEaseFC software.

### Real-Time Quantitative RT-PCR

Total mRNA was isolated from SH-SY5Y cells or mouse brain tissues using TRIzol reagent, and mRNA levels were analyzed using real-time quantitative RT-PCR with the Bio-Rad iCycler system. The primers for target genes used in this study were synthesized from Biosune Tech., Co., Ltd. (Shanghai). The sequences are as follows: HDAC6: forward, GGAAAAGGTCGCCAGAAACTT and reverse, GGCCGG TTGAGGTCATAGTT; NLRP3: forward, ACGAGTCCTGGTG ACTTGT and reverse, GTCCACACAGAAAGTTCTCTT AGC; IL-1β: forward, TGCCACCTTTTGACAGTGATG and reverse, TGATGTGCTGCTGCGAGATT; IL-18: forward, AC TTTGGCCGACTTCACTGT and reverse, GGGGTTCACT GGCACTTTGA; IL-6: forward, GATCAAAGTGCCAG TGAACCC and reverse, GGTCACAGCCAGTCCTCTTA; GAPDH: forward, TACCCGGACTGGATTCTACG and reverse, AAGTTGGTGGGCTGTCAATC. Gene expression was calculated using the cycle threshold (Ct) values according to the ^ΔΔ^Ct method.

### Immunoprecipitation

Immunoprecipitation was performed based on a previous study (An et al., 2008). Cell extracts were homogenized in NP-40 buffer with protease inhibitors. Samples containing the same amount of protein were incubated with primary antibody Prx2 (1 μg, 10545-2-AP, ProteinTech) and then with 30 μl of protein A/G agarose beads (P2012, Beyotime) at 4°C to allow formation of antigen–antibody–beads complex. The beads complex was collected after centrifugation at 2500 rpm for 5 min and washed five times with ice-cold buffer and were then boiled with SDS sample buffer for 10 min. After centrifugation at 11,000 rpm for 1 min, the supernatants were separated on 12% SDS-PAGE, and the protein levels were analyzed using acetylated lysine primary antibody and Prx2 primary antibody by Western blotting.

### Reactive Oxygen Species (ROS) Measurement

Cellular ROS were assessed using the dichloro-dihydro-fluorescein diacetate (DCFH-DA) fluorescence assay (Jing et al., 2015). According to the manufacturer’s instruction, cells were incubated with fresh DMEM-F12 medium containing 10 μM DCFH-DA (D6470-25, Sigma-Aldrich) at 37°C for 30 min and then washed with PBS buffer. After trypsinization, the cells were centrifugated at 1000 rpm for 5 min. Then, the levels of cellular ROS were measured by flow cytometry.

### Cell Viability Assessment

Cells were pretreated with TBA at indicated concentrations for 2 h and were then exposed to 100 μM 6-OHDA for additional 24 h to assess the effect of TBA on cell viability. Cell viability was measured using the CCK-8 assay according to the manufacturer’s instruction (CK-04, DOJINDO). Briefly, cells were incubated with 10 μl of CCK-8 solution at 37°C for 30 min and then the absorbance was measured at 450 and 600 nm on a plate reader.

### TUNEL Staining

TUNEL staining was performed to detect apoptotic cells using the *in situ* cell death detection kit (12156792910, Roche Diagnostic). Briefly, the slides were fixed with 4% paraformaldehyde at 4°C for 1 h, and incubated in 3% H_2_O_2_ for 10 min. After incubation in 0.1% Triton X-100 for 15 min, the slides were incubated in TUNEL reaction mixture at 37°C for 1 h. Finally, the slides were counterstained with DAPI and observed by fluorescence microscope.

### Apoptosis Assay

The number of apoptotic cells was measured using the propidium iodide (PI)/Annexin V-FITC apoptosis detection kit (FXP018-100, 4A Biotech) with flow cytometry according to the manufacturer’s instruction. Briefly, cells were treated with TBA for 2 h before being exposed to 6-OHDA for an additional 24 h. The cells were harvested, washed with ice-cold PBS, and then evaluated for apoptosis by double staining with PI and annexin V-FITC in binding buffer using a Cytoflex flow cytometer.

### Immunohistochemistry and Immunofluorescence Staining

Mice were anesthetized with sodium pentobarbital, transcardially perfused with normal saline, and followed by 4% paraformaldehyde in 0.1M PBS. Brains were dissected and cryopreserved in 30% sucrose for 48 h. After freezing, the brains were coronally sectioned as described previously (Zhang et al., 2008). For immunohistochemistry, samples were incubated with TH antibody (MAB318, Millipore) followed by horseradish peroxidase (HRP)-conjugated secondary antibody. The sections were visualized using diaminobenzidine (DAB). Digital images were collected in bright-field microscope. For immunofluorescence staining, samples were incubated with GFAP antibody (MAB360, Millipore) and IBA1 antibody (MABN92, Millipore) to observe glial cell proliferation or with 4-HNE antibody (ab46545, Abcam) to evaluate ROS levels and then incubated with Alexa Fluor 488 anti-rabbit IgG (Invitrogen) or 594 anti-mouse IgG (Invitrogen) secondary antibodies and digital images were collected in fluorescence microscope.

Assessment of the total number of TH^+^ cells in the SNpc was made according to the optical fractionator probe (MicroBrightfield) by an investigator blinded to the experimental design. Every fifth section covering the entire extent of these regions was included in the counting procedure. The data were expressed as a percentage of the corresponding area from the non-injected, intact side. Density of TH^+^ fibers was measured by densitometry in the striatum using the Image J software, the data are expressed as a percentage of the corresponding area from the intact side.

### HPLC Analysis

Striatal dopamine (DA) and its metabolites, including homovanillic acid (HVA) and dihydroxyphenylacetic acid (DOPAC), were measured using the high-performance liquid chromatography–tandem mass spectrometry (HPLC-MS/MS) method. Briefly, striata were individually weighed and homogenized in 0.5M formic acid with a concentration of 10 μl/1 mg tissue. After centrifugation for 30 min at 4°C, the supernatants were filtered with 0.22 μM filter membrane and analyzed according to an established protocol (Ren et al., 2018).

### Apomorphine-Induced Rotation Test

Apomorphine-induced rotations were monitored over 3 weeks’ time, starting from 1 week post 6-OHDA lesion according to previous published protocols (Signore et al., 2006). Apomorphine was subcutaneously injected into mice at a dose of 0.1 mg/kg (Sigma), with mice placed individually in plastic beakers (diameter: 13 cm), and videotaped from above for 30 min. Quantitative analyses of completed (360°) left and right rotations were made offline by an investigator blinded to the experimental conditions.

### Statistical Analyses

Data are expressed as mean ± SEM. The significance of differences in mean values between or within multiple groups was examined by one-way or two-way ANOVA followed by Bonferroni *post hoc* test. A *p*-value of < 0.05 was considered statistically significant.

## Results

### TBA Inhibits 6-OHDA-Induced Activation of NLRP3 Inflammasome in SH-SY5Y Cells

**FIGURE 2:**
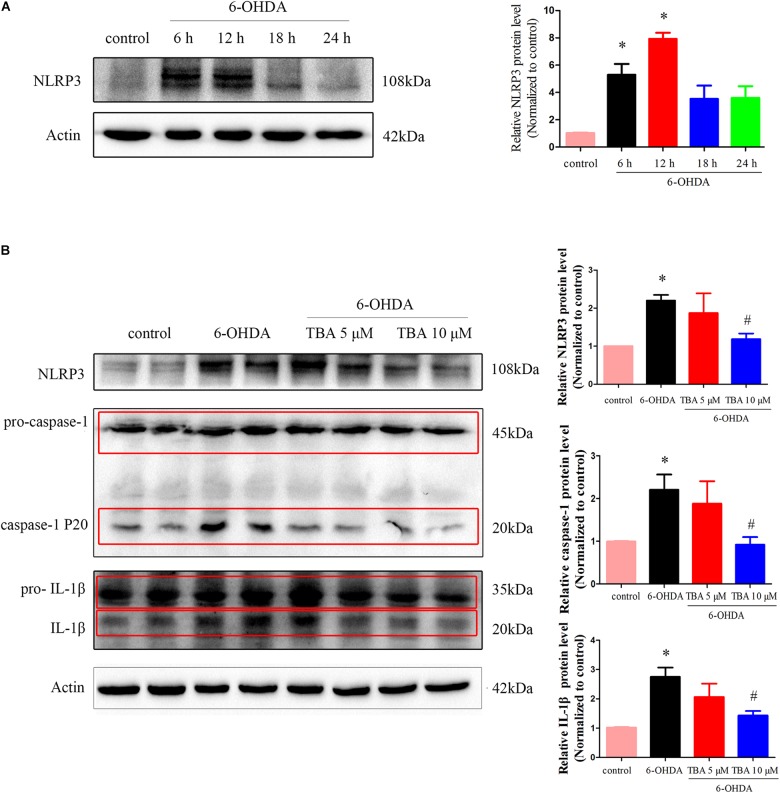
Pharmacological inhibition of HDAC6 with TBA inhibits NLRP3 inflammasome activation induced by 6-OHDA in SH-SY5Y cells. **(A)** Representative Western blotting and summarized data showing protein levels of NLRP3 in SH-SY5Y cells at different time points after 6-OHDA injury. **(B)** Representative Western blotting and summarized data showing the effects of TBA on expressions of NLRP3, pro-caspase-1, activated caspase-1 (caspase-1 P20), pro-IL-1β, and activated IL-1β in SH-SY5Y cells after 6-OHDA injury. Data are presented as mean ± SEM of three independent experiments. One-way ANOVA followed by Bonferroni *post hoc* test was used to assess significant differences among groups. *p < 0.05 vs. control; *^#^p* < 0.05 vs. the 6-OHDA-treated group.

As shown in Figure 2A, we detected protein levels of NLRP3 at different time points after 6-OHDA injury in SH-SY5Y cells, and the expression of NLRP3 was increased at 6 and 12 h. TBA was used to examine whether deacetylation activity of HDAC6 regulates NLRP3 activation in this study. As shown in Figure 2B, TBA partly attenuated 6-OHDA-induced increased expression of NLRP3 and activated caspase-1 and IL-1β. These results indicate that 6-OHDA-induced NLRP3 activation is associated with deacetylation activity of HDAC6, and pharmacological inhibition of HDAC6 can attenuate NLRP3 activation.

### TBA Protects SH-SY5Y Cells From 6-OHDA-Induced Injury

**FIGURE 3:**
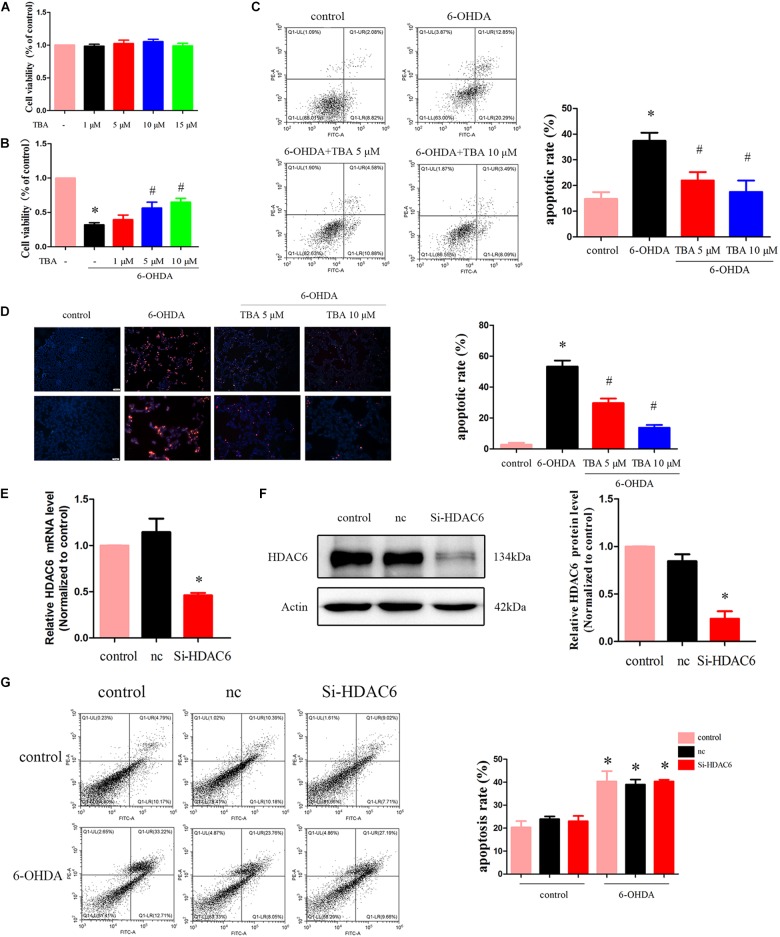
TBA attenuates 6-OHDA-induced cell death. **(A)** SH-SY5Y cells were incubated with TBA at indicated concentrations for 24 h, and cell viability was determined by CCK-8 assay. **(B)** Cells were pretreated with TBA for 2 h before exposure to 100 μM 6-OHDA for an additional 24 h. The effect of TBA on cell viability was measured by CCK-8 assay. **(C)** The effect of TBA on cell apoptosis was measured by Annexin-V/PI staining. The apoptotic rate calculated from the flow cytometry data was also shown. **(D)** The effects of TBA on apoptotic cells were determined by the TUNEL assay. **(E)** Real-time RT-PCR analysis of HDAC6 mRNA levels in siRNA-HDAC6-transfected cells. **(F)** Representative Western blotting and summarized data showing the relative HDAC6 expression in siRNA-HDAC6-transfected cells. **(G)** The effect of siRNA-HDAC6 on cell apoptosis was measured by Annexin-V/PI staining. Data are presented as mean ± SEM of three independent experiments. One-way ANOVA followed by Bonferroni *post hoc* test was used to assess significant differences among groups. *p < 0.05 vs. control; *^#^p* < 0.05 vs. the 6-OHDA-treated group.

We further determined the potential protective role of pharmacological inhibition of HDAC6 with TBA in 6-OHDA-induced cell death. We first assessed the effect of TBA itself on cell viability. As shown in Figure 3A, TBA (up to 15 μM) had no obvious effects on the growth of SH-SY5Y cells. We then investigated the effect of TBA on cell viability in SH-SY5Y cells treated with 6-OHDA. As shown in Figure 3B, TBA showed a dose-dependent protective effect on cell viability. Subsequently, we used PI/Annexin V-FITC staining and flow cytometry to analyze cell apoptosis. It was found that 6-OHDA significantly increased cell apoptosis, but pretreatment with TBA decreased the apoptotic rate (Figure 3C). The anti-apoptotic effect of TBA was further confirmed by the TUNEL assay (Figure 3D). As we know, TBA only targets the deacetylase catalytic domain but has no effects on other domains of HDAC6; therefore, we further examined whether the protective effects of TBA are due to pharmacological inhibition of HDAC6 by the approach of gene silence of HDAC6. As shown in Figures 3E,F, HDAC6 levels were decreased in siRNA-HDAC6-transfected cells. Flow cytometry revealed that gene silence of HDAC6 had no obvious effects on 6-OHDA-induced cell death, as shown in Figure 3G. These results suggest that pharmacological inhibition on the deacetylation activity of HDAC6 is involved in TBA-induced protective effects in 6-OHDA-exposed SH-SY5Y cells.

### TBA Suppresses NLRP3 Inflammatory Response in the Nigrostriatal System in the 6-OHDA-Induced PD Mouse Model

**FIGURE 4:**
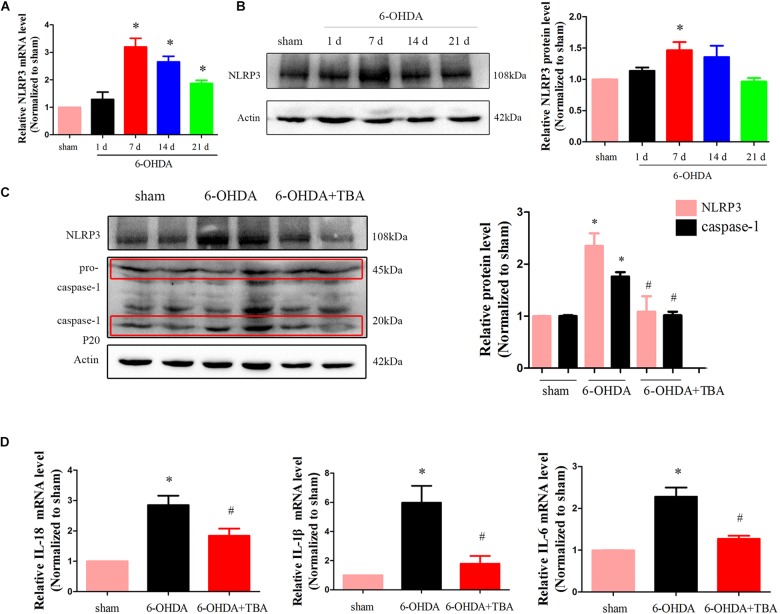
TBA suppresses NLRP3 inflammatory response in the nigrostriatal system in the 6-OHDA-induced PD mouse model. **(A)** Real-time RT-PCR analysis of NLRP3 mRNA at different time points after 6-OHDA injury. **(B)** Representative Western blotting and summarized data showing protein levels of NLRP3 at different time points after 6-OHDA injury. **(C)** Representative Western blotting and summarized data showing the effects of TBA on protein levels of NLRP3 and caspase-1 after 6-OHDA injury. **(D)** Real-time RT-PCR analysis showing the effects of TBA on IL-1β, IL-18, and IL-6 after 6-OHDA injury. Data are presented as mean ± SEM. Differences among groups were assessed by one-way ANOVA followed by Bonferroni’s test. *p < 0.05 vs. sham-operated mice; *^#^p* < 0.05 vs. the 6-OHDA-treated group (*n* = 5).

**FIGURE 5:**
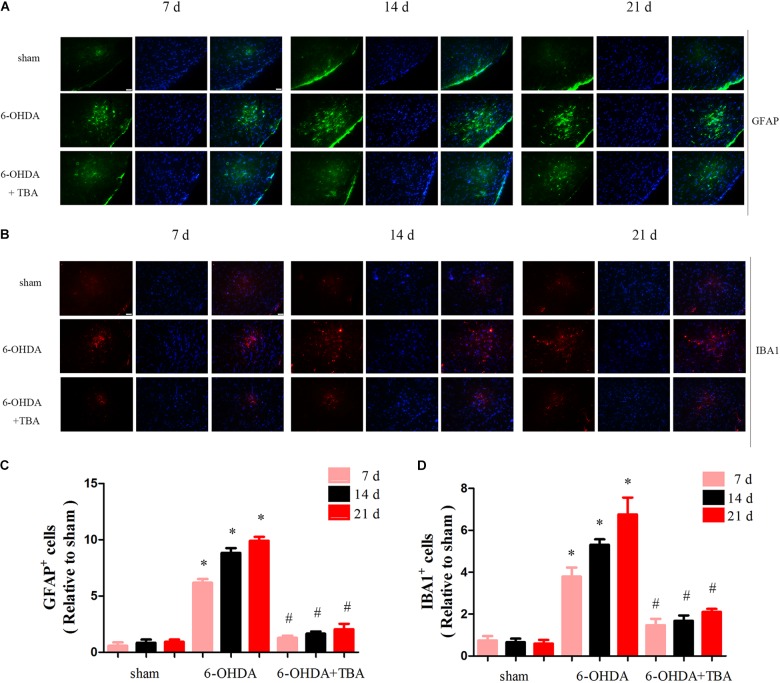
TBA ameliorates 6-OHDA-induced glial cell proliferation in the SNpc area. **(A,C)** Immunofluorescence for GFAP-positive astrocytes (green) and DAPI (blue) showing that TBA significantly decreased 6-OHDA-induced astrocytic activation. **(B,D)** Immunofluorescence for IBA1-positive microglia (red) and DAPI (blue) showing that TBA significantly decreased 6-OHDA-induced microglial activation. Data are presented as mean ± SEM. Two-way ANOVA followed by Bonferroni’s test were used to assess differences among groups. *p < 0.05 vs. sham-operated mice; *^#^p* < 0.05 vs. the 6-OHDA-treated group (*n* = 4).

As shown in Figure 4A, we assessed mRNA levels of NLRP3 in the nigrostriatal tissue at different time points after 6-OHDA injection, and NLRP3 mRNA expressions were high from 7 to 21 days. Western blotting further confirmed the increased level of NLRP3 (Figure 4B). Then, TBA was used to examine whether the NLRP3 inflammatory response can be regulated by the deacetylation activity of HDAC6. As shown in Figures 4C,D, TBA inhibited NLRP3 activation, as demonstrated by decreased expressions of NLRP3, matured caspase-1, and inflammatory cytokines. Studies have demonstrated that NLRP3 has functional importance in astrocytic and microglial activation (Freeman et al., 2017). Therefore, we examined whether TBA could prevent glial cell activation. As shown in Figures 5A,B, the number of astrocytes (astrocyte marker GFAP) and microglia (microglia marker IBA1) in the SNpc was increased in 6-OHDA-exposed mice in a time-dependent manner at 7, 14, and 21 days after 6-OHDA injection. Compared with 6-OHDA-alone group, treatment with TBA markedly attenuated astrocytes and microglia activation, as shown by a reduced density of GFAP and IBA1 (Figures 5C,D). These findings indicate that pharmacological inhibition on the deacetylation activity of HDAC6 could reduce NLRP3 inflammatory responses in the 6-OHDA-induced PD mouse model.

### TBA Attenuates Dopaminergic Neurotoxicity in the 6-OHDA-Induced PD Mouse Model

**FIGURE 6:**
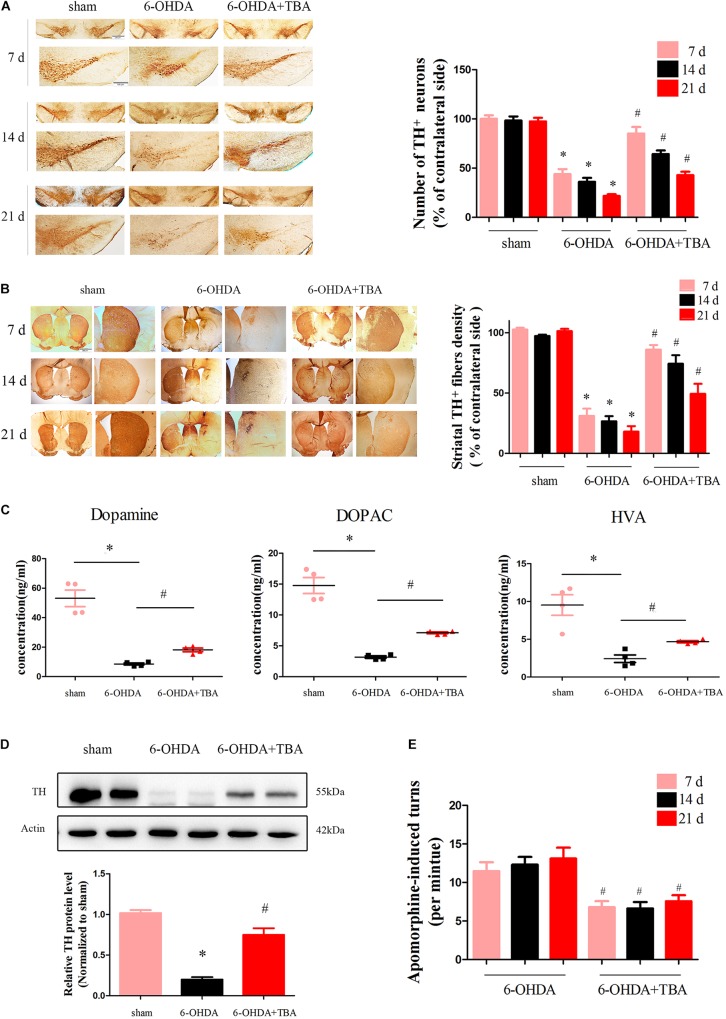
TBA attenuates 6-OHDA-induced dopaminergic neurotoxicity *in vivo*. **(A,B)** TH immunoreactivity in the STR and SNpc at different time points after 6-OHDA injection. Data are presented as mean ± SEM. *p < 0.05 vs. sham-operated mice; *^#^p* < 0.05 vs. the 6-OHDA-treated group (*n* = 5). Differences among groups were assessed by two-way ANOVA followed by Bonferroni’s test. **(C)** Striatal levels of DA and its metabolites measured by HPLC analysis. **(D)** Representative immunoblot documents and summarized data showing that TBA attenuates 6-OHDA-induced loss of TH in mice striatal tissues. **(E)** Apomorphine-induced turns were assessed at 7, 14, and 21 days after 6-OHDA injection. Data are presented as mean ± SEM. *p < 0.05 vs. sham-operated mice; *^#^p* < 0.05 vs. the 6-OHDA-treated group (*n* = 5) according to a one-way ANOVA followed by Bonferroni’s test.

To determine if TBA treatment protects the nigrostriatal system, brains were collected to examine the levels of TH, an important enzyme in dopaminergic neurons. Immunohistochemical analysis showed that 6-OHDA-induced loss of TH-positive neurons and fibers in the SNpc and STR was significantly attenuated by TBA treatment (Figures 6A,B). These results were further supported by immunoblots of striatal extracts evaluated using an anti-TH antibody, which showed higher TH protein levels in TBA-treated mice (Figure 6D). We also measured striatal levels of DA, DOPAC, and HVA and found that the reduction in DA and its metabolites after 6-OHDA injury was recovered by TBA treatment (Figure 6C). In order to correlate early biochemical changes with long-term motor alterations induced by 6-OHDA, mice were monitored for apomorphine-induced rotation at 7, 14, and 21 days after 6-OHDA lesion. Apomorphine-induced asymmetrical rotations contralateral to the 6-OHDA injection site were significantly reduced by TBA treatment as compared to mice injured with 6-OHDA alone (Figure 6E). Collectively, these findings indicate that TBA has protective effects in the nigrostriatal system.

### TBA Recovers Acetylation of Prx2 and Reduces ROS

**FIGURE 7:**
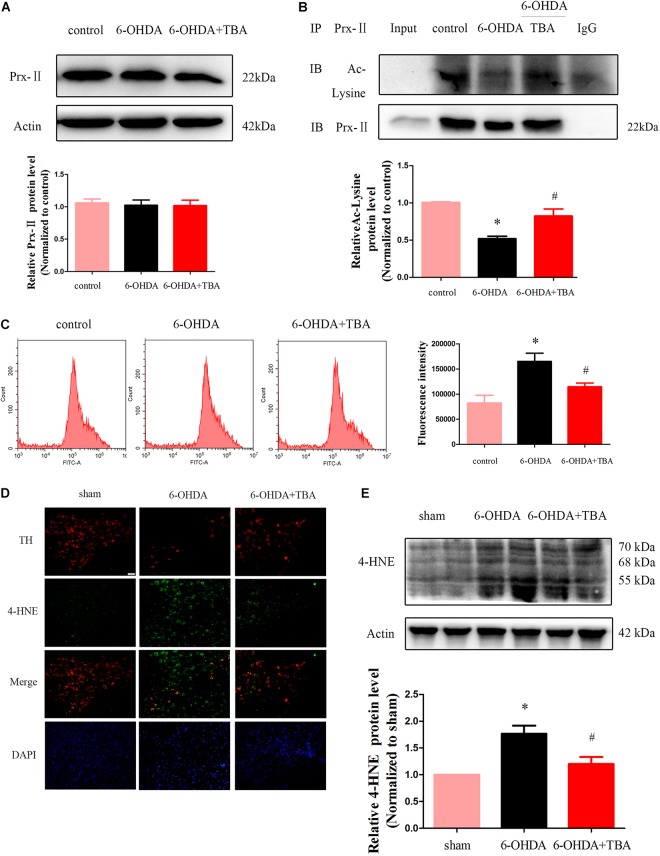
TBA recovers acetylation of Prx2 and reduces ROS. **(A)** Representative Western blotting and summarized data showing protein levels of Prx2 in SH-SY5Y cells after different treatments. **(B)** Immunoprecipitation and Western blotting showing the acetylation levels of Prx2 in SH-SY5Y cells after different treatments. **(C)** Intracellular levels of ROS were measured with DCFH-DA staining and flow cytometry. **(D)** Immunofluorescent staining showing expressions of 4-HNE in the SNpc after different treatments. **(E)** Representative Western blotting and summarized data showing the protein level of 4-HNE in the STR. Data are presented as mean ± SEM. Differences among groups were assessed by one-way ANOVA followed by Bonferroni’s test. *p < 0.05 vs. control or sham; *^#^p* < 0.05 vs. the 6-OHDA-treated group (*n* = 4).

Oxidative stress is known to activate the NLRP3 inflammasome (Martinon, 2010), and our previous *in vivo* study found that TBA decreased ROS production. Thus, we speculated that TBA-induced anti-NLRP3 inflammatory effect is partly associated with ROS reduction. Accordingly, we investigated whether TBA regulates acetylation of Prx2 and ROS production in SH-SY5Y cells. As shown in Figures 7A,B, the acetylation levels of Prx2 were decreased after 6-OHDA injury but were significantly recovered by TBA treatment. ROS production was detected by DCFH-DA staining and flow cytometry. As shown in Figure 7C, ROS were significantly higher after 6-OHDA injury, but TBA treatment reduced ROS levels. In addition, we examined ROS levels in the nigrostriatal area. Our results showed that the levels of 4-HNE, a marker to evaluate oxidative stress (Ye et al., 2019), were dramatically higher after 6-OHDA injury but were remarkably reduced by TBA treatment, as shown in Figures 7D,E. These findings indicate that TBA increases acetylation of Prx2 and thus reduces oxidative stress.

## Discussion

In the present study, TBA, a specific pharmacological inhibitor of HDAC6, was used to test the hypothesis that inhibition of deacetylation activity of HDAC6 represents a novel strategy for decreasing NLRP3 inflammatory response in PD models. We found that TBA treatment significantly inhibited 6-OHDA-induced NLRP3 activation, as demonstrated by decreased expressions of NLRP3 and matured caspase-1 and IL-1β. These effects coincided with the inhibitory effects of TBA on glial cell proliferation. Moreover, administration of TBA reduced dopaminergic neuronal degeneration, recovered acetylation levels of Prx2, and decreased ROS production. The findings suggest that pharmacological inhibition of HDAC6 with TBA suppresses NLRP3 activation and attenuates dopaminergic neurotoxicity, partly due to decreased oxidative stress caused by Prx2 acetylation. This study is the first to demonstrate that the deacetylation catalytic domain of HDAC6 may serve as a potential target for regulating the NLRP3 inflammatory response in PD.

**FIGURE 8:**
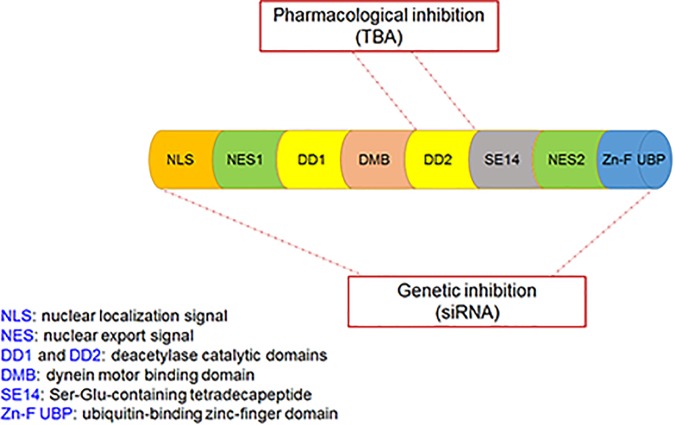
The functional domains of the HDAC6 protein and the target sites of pharmacological inhibition and genetic inhibition of HDAC6. Pharmacological inhibition of HDAC6 with TBA targets DD2 only, whereas genetic inhibition of HDAC6 with siRNA targets all domains.

The effects of non-enzyme activity (i.e., ubiquitin-binding activity) of HDAC6 on PD pathogenesis have been reported (Du et al., 2010), but the role of its enzymatic activity (i.e., the deacetylation activity) on PD pathogenesis remains unclear. This study demonstrated the protective effects of HDAC6 inhibitors in PD models; the findings are not consistent with those in previous studies using HDAC6-knockout animals (Du and Jiao, 2011). In a study using *Drosophila* models of PD, HDAC6 was found to promote inclusion formation, leading to elimination of aggregates and suppression of dopaminergic cell loss, indicating that HDAC6 itself may play a protective role in cell injury (Du et al., 2010). The present study also found that gene silence of HDAC6 did not protect dopaminergic cells from death. The discrepancy between the experimental observations could be explained by different treatment approaches of HDAC6: specific pharmacological inhibitors and the genetic knockout approach. As mentioned above, HDAC6 has unique structural properties, which provide support for its broad functions in mediating cell response. Both deacetylase catalytic domains (DD1 and DD2) of HDAC6 contribute to its enzymatic activity, i.e., deacetylating some cytoplasmic proteins, and ZnF-UBP exerts non-enzymatic activity, i.e., sensing the ubiquitinated misfolded proteins and promoting inclusion formation and aggregate degradation. To date, all specific HDAC6 inhibitors have been shown to target DD2 and only regulate the deacetylation activity (Batchu et al., 2016); however, the genetic knockout approach could target all domains, thus affecting all functions of the protein (Figure 8).

NLRP3, a member of Nod-like receptor family, forms a cytosolic multiprotein complex with ASC and pro-caspase-1, known as inflammasome, to regulate activation of IL-1β and IL-18 (Mangan et al., 2018). NLRP3 plays a vital role in autoimmune diseases, and NLRP3-induced maturation of pro-inflammatory cytokines is implicated in PD pathogenesis (Martinez et al., 2017). Given the role of HDAC6 specific inhibitors in inflammatory conditions (de Zoeten et al., 2011; Di Liddo et al., 2016; Chang et al., 2018; Karki et al., 2019; Ran and Zhou, 2019), we examined whether NLRP3 is a potential molecular target of HDAC6 for the regulation of cellular events in PD. We found that NLRP3 was significantly activated after 6-OHDA injury but could be suppressed by TBA, and TBA alleviated glial cell proliferation. Although the mechanisms underlying the TBA effect on NLRP3 activation are not clearly understood, our findings suggest that Prx may be a potential target. The Prx family, comprising Prx1, 2, 3, 4, 5, and 6, belongs to the redox regulatory proteins, which maintain the intracellular reducing milieu (Detienne et al., 2018). Prx1 and Prx2 are specific targets of HDAC6 (Parmigiani et al., 2008), and once deacetylated, Prx1 and Prx2 lose their antioxidant capacity, resulting in free radical accumulation. Increasing evidence has indicated that the deacetylated form of Prx1 or Prx2 contributes to oxidative stress in neurological diseases (Choi et al., 2017; Jian et al., 2017; Leyk et al., 2017). ROS have been known to activate the NLRP3 inflammasome (Sorbara and Girardin, 2011; Shridas et al., 2018), and therapeutic strategies to lessen oxidative stress can inhibit inflammatory injury by attenuating ROS-induced NLRP3 activation (Liu et al., 2018; Zhang et al., 2018). In the present study, we found that acetylation levels of Prx2 were decreased after 6-OHDA injury, and TBA treatment recovered Prx2 acetylation and reduced ROS production. It is noted that our current research does not directly evaluate possible off-target effects of TBA. However, our data are supported by growing evidence showing that TBA can effectively ameliorate pathological damages in animal models of neurodegenerative diseases, and these actions appear to be attributable to restoration of impaired substrate acetylation (Shen and Kozikowski, 2020). In line with these results, our study shows that TBA at a commonly used dose results in a marked increase in Prx2 acetylation, reverses NLRP3 inflammatory response, and protects dopaminergic neurons against 6-OHDA-induced damages. Indeed, like our observations, other studies have also reported that TBA and genetic inhibition of HDAC6 may produce divergent effects (Bobrowska et al., 2011; Guedes-Dias et al., 2015). We propose that phenomenon may be related to the fact that TBA specifically targets the deacetylase catalytic domain-DD2 of HDAC6, whereas genetic approaches affect both of the deacetylase domains as well as the Zn-F UBP (Figure 8). Therefore, we speculate that TBA-induced Prx2 acetylation may lead to its anti-NLRP3 inflammatory effects; or alternatively, it is possible that TBA directly acetylates NLRP3, affecting the inflammasome stability.

## Conclusion

We think that the specificity of TBA to the deacetylase catalytic domain of HDAC6, i.e., the restoration effect of TBA on impaired substrate acetylation caused by HDAC6, contributes to its pharmacological effects in our study. Furthermore, it would help to clarify the specificity of TBA if we also check other pharmacological inhibition of HDAC6, such as ACY-1215.

## Data Availability Statement

The raw data generated for this study are available on request to the corresponding author.

## Ethics Statement

The animal study was reviewed and approved by the Experimental Animal Ethics Committee of Shandong University.

## Author Contributions

BZ and HL planned experiments, interpreted data, and approved the manuscript for publication. BZ wrote the article. SY performed most of the experiments and analyzed data. XW participated in the animal experiment. WJ and SZ participated in the experiment design and data interpretation. YQ and JL participated in the cell experiment. FJ participated in the experiment design and manuscript revision. All authors read and approved the final article.

## Conflict of Interest

The authors declare that the research was conducted in the absence of any commercial or financial relationships that could be construed as a potential conflict of interest.
